# Design and Evaluation of a Flexible Substrate-Based Microstrip Sensor for Partial Discharge Detection in High-Voltage Equipment

**DOI:** 10.3390/s26113304

**Published:** 2026-05-22

**Authors:** Shuhao Dong, Xiao Hu

**Affiliations:** College of Electrical Engineering, Guizhou University, Guiyang 550025, China; gs.shdong23@gzu.edu.cn

**Keywords:** cable joint, GTEM cell, microstrip antenna, partial discharge (PD), power transformer, whale optimization algorithm (WOA)

## Abstract

**Highlights:**

**What are the main findings?**

- A flexible microstrip antenna with beveled meandering and a partial ground plane broadens its bandwidth from 0.612–0.625 GHz to 0.346–2.0 GHz while shrinking its footprint to 75.3% of its original size.
- The improved whale optimization algorithm (I-WOA), which combines Sobol sequence initialization with Q-learning, efficiently optimizes the antenna’s structural parameters for simultaneous bandwidth maximization and size minimization.

**What are the implications of the main findings?**

- The novel sensor overcomes the installation rigidity of conventional microstrip antennas by enabling non-invasive, broadband RF detection of partial discharges in both power transformers and cable joints.
- The practical advantage of a flexible substrate for curved equipment surfaces is evidenced by a 14% increase in response amplitude when the antenna is conformally wrapped around a cable joint.

**Abstract:**

Partial discharge (PD) detection effectively identifies insulation defects in power equipment. Radio frequency (RF) methods for PD detection offer promising advantages due to their non-invasive measurement capability and ability to locate discharge sources. However, microstrip antennas used as RF sensors for PD detection suffer from narrow bandwidth and limited installation flexibility. To address these limitations, this paper presents a novel flexible microstrip antenna design. By incorporating a partial ground plane and oblique-cut meandering techniques and optimizing the structural parameters using an improved whale optimization algorithm (I-WOA), the operating bandwidth is expanded from 0.612–0.625 GHz to 0.346–2.0 GHz, while the overall size is reduced to 75.3% of its original dimensions. The antenna’s performance was validated through GTEM cell measurements and PD calibration pulse tests, confirming its suitability for RF detection of PD in power equipment such as transformers and cable joints. Notably, when the antenna was conformally wrapped around a cable joint, the response amplitude increased by 14%. This study contributes to the development of a low-cost, broadband, and flexibly installable RF sensor for partial discharge detection.

## 1. Introduction

Electrical equipment in power systems is subjected to electric, thermal, mechanical, and environmental stresses, leading to insulation aging and degradation that may cause partial discharge (PD) and eventually equipment failure. PD generates multiple physical phenomena such as transient currents, sound and light emissions, electromagnetic (EM) radiation, gaseous byproducts, etc. Correspondingly, various techniques have been developed to measure PD, including the conventional method as specified in IEC 60270 [1], and non-conventional methods such as ultrasonic measurement [2], radiometric measurement [3], gas chromatography [4], etc. Among these, radiometric measurement uses radio frequency (RF) techniques to measure PD-induced EM waves at frequencies up to 3000 MHz, and is sometimes referred to as RF detection [5]. Compared with the conventional IEC method, RF detection is preferred in on-site PD measurement because it can achieve higher signal-to-noise ratios under on-site conditions and allows non-contact detection [6]. However, since the spectral characteristics of PD-induced EM signals and sensor mounting conditions can vary significantly across different types of electrical equipment, RF PD sensors may need to be designed individually for specific applications. For example, EM signals generated by internal PD of oil-immersed power transformers have significant spectral content in the 500–1500 MHz frequency range [7]. RF sensors including planar disk, spiral, and horn antennas with suitable operating frequency ranges have been developed and applied to PD detection in transformers [8]. In the case of power cables, cable accessories (including joints and terminations), especially those manufactured on site, can contain defects that may cause PD after the cables are put into operation. Solutions for on-site PD measurement of cables and cable accessories have predominantly used high-frequency current transformers (HFCTs) as PD sensors [9]. EM signals excited by PD in cable joints can have evident spectral content up to 600 MHz [10]. However, RF detection methods have been explored relatively less for this application, probably because there is no RF sensor as widely recognized as HFCTs.

Microstrip antennas offer advantages such as a simple structure, compact size, and tunable bandwidth [11], and have thus attracted considerable attention in research on RF sensors for PD detection. Xavier et al. developed a circular microstrip antenna using a truncated ground plane, achieving an operating bandwidth of 0.305–1.495 GHz and an average gain of 4.92 dBi. However, the antenna was relatively large (300 × 300 × 1.52 mm) and employed a rigid FR4 substrate, lacking installation flexibility [12]. Du et al. designed an antenna based on a hollow circular fractal structure, which exhibited a bandwidth of 0.5–1.06 GHz and an average gain of 3.8 dBi, with dimensions of 150 × 180 × 1.6 mm. Although this design achieved miniaturization, its bandwidth did not cover the full frequency range required for UHF detection of transformer partial discharges (500–1500 MHz), and it still relied on a rigid substrate [13]. In recent years, the application of flexible antennas in PD detection has gradually gained attention. Zhang et al. employed flex-print technology to realize a flexible planar monopole antenna with dimensions of 142 × 195 × 0.28 mm; however, its bandwidth exhibited a discrete narrowband distribution (0.57–0.83 GHz, 1.38–1.8 GHz, and 2.2–2.76 GHz), which is unfavorable for broadband PD detection [14]. Taher et al. [15] systematically reviewed the use of flexible substrates—including polyimide, PDMS, and Rogers—in UHF antennas and noted that flexible conformal antennas hold significant promise for PD detection in high-voltage equipment. Nevertheless, research on flexible antennas for curved equipment such as cable joints remains limited, and few studies have concurrently validated antenna applicability across multiple types of power equipment. As evidenced by the above studies, existing microstrip antenna sensors each emphasize different aspects—such as bandwidth, size, flexibility, and frequency coverage—but struggle to simultaneously fulfill all these requirements. Specifically, the antenna reported in [12] offers wide bandwidth and high gain but suffers from large size and lack of flexibility; the design in [13] is compact yet fails to fully cover the UHF detection band for transformers; the flexible antenna in [14] achieves flexibility at the cost of discontinuous bandwidth and detection blind spots; and the review by Taher et al. [15] acknowledges the potential of flexible antennas for PD detection while noting that practical application research remains insufficient. Consequently, developing a microstrip antenna that integrates broadband operation, miniaturization, and flexibility, and validating its applicability across various power equipment, holds significant engineering value.

The remainder of this paper is organized as follows. Section 2 presents the fundamental configurations of the microstrip antenna and a study of essential geometric parameters influencing its performance. Section 3 describes the improved whale optimization algorithm (I-WOA) and its application to determining optimal geometric parameters. Section 4 reports on the fabricated prototype sensor and experiments conducted to characterize its bandwidth and sensitivity. Section 5 demonstrates the performance of the developed sensor through PD calibration pulse measurements on a transformer and a cable joint.

## 2. Configurations of the Microstrip Sensor

**Figure 1:**
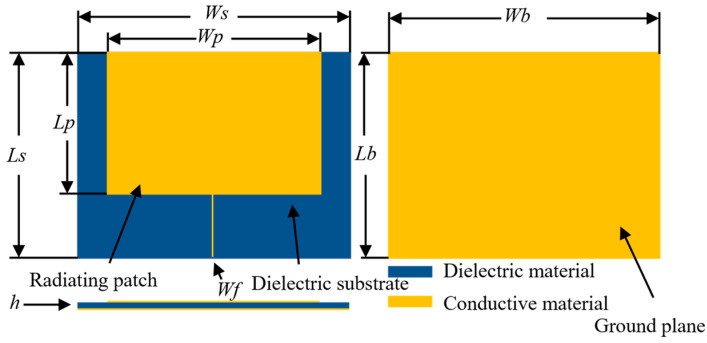
Structure of a microstrip antenna.

The flexible microstrip antenna features a multilayer structure, comprising a radiating patch, a dielectric substrate, and a ground plane, as illustrated in Figure 1. Its flexibility is primarily attributable to the use of polyimide as the dielectric substrate material, which has a relative permittivity of 3.5 and a thickness of 0.25 mm. Polyimide not only offers excellent electrical insulation but also exhibits good mechanical ductility, enabling the antenna to maintain structural integrity and stable performance across diverse application environments. Based on the selected substrate parameters, the initial dimensions of a conventional flexible microstrip antenna were estimated using the method described in Reference [16]; the results are presented in Table 1.

**Figure 2:**
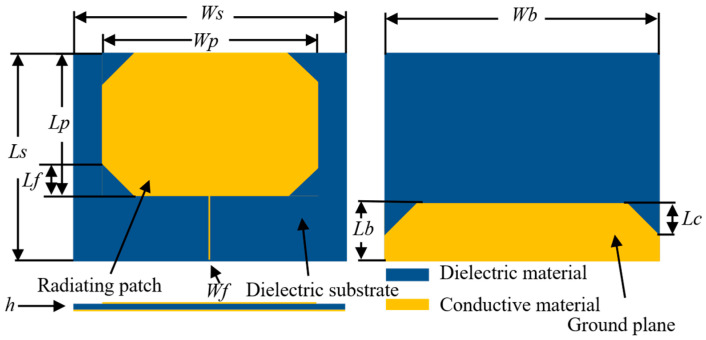
Structure of the novel flexible microstrip antenna.

The flexible microstrip antenna shown in Figure 1 with the dimensions listed in Table 1 was modeled and simulated using the high-frequency electromagnetic simulation software HFSS (Ansys Electronics Desktop 2023 R1). The results revealed an operating bandwidth of only 0.612–0.625 GHz, which fails to meet the wideband requirements for partial discharge (PD) signal detection. To address this narrowband issue, this paper employs a partial ground plane and a meandering technique to improve the antenna structure. The partial ground plane effectively suppresses radiation loss, thereby extending the operating bandwidth. The meandering technique lengthens the current propagation path by bending the current on the radiating patch, thus broadening the bandwidth without increasing the antenna dimensions. Conventional meandering methods typically involve cutting rectangular or trapezoidal slots in the patch. In this work, meandering is realized by cutting right-angled triangular bevels at the four corners of the patch. The resulting bevels alter the surface current distribution, exciting additional resonance modes, while also creating a gradually varying spacing between the lower edge of the radiating patch and the feed line. This configuration facilitates impedance matching and promotes the merging of multiple resonant frequencies, thereby achieving broadband performance while maintaining a compact antenna footprint. The geometry of the improved antenna is depicted in Figure 2.

**Figure 3:**
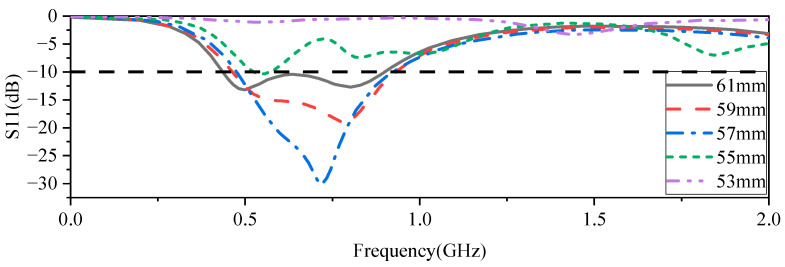
Partial ground lengths on the reflection coefficient of microstrip antennas.

To investigate the influence of ground plane size on bandwidth, simulations were performed for various ground plane lengths; the results are presented in Figure 3. The simulated data indicate that when the ground plane length is slightly shorter than the feed line length, the antenna bandwidth improves markedly. Specifically, with a ground plane length of 57 mm, the antenna achieves a bandwidth of 0.475–0.923 GHz.

**Figure 4:**
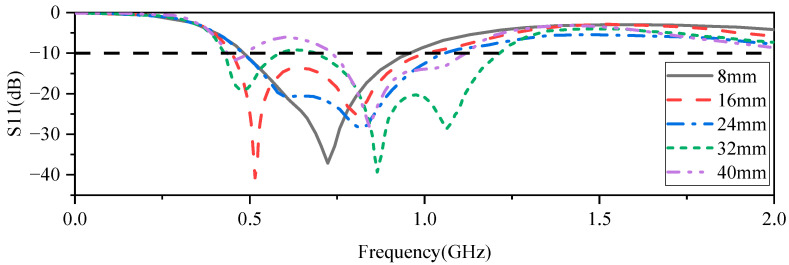
Radiating patch truncation lengths on the reflection coefficient of microstrip antennas.

With the ground plane length maintained at 57 mm, right-angled triangular bevels were introduced at the four corners of the radiating patch. The influence of different bevel lengths on the reflection coefficient was evaluated through simulations, and the results are presented in Figure 4. Compared with the unbeveled case (Figure 3, bandwidth 0.475–0.923 GHz), a bevel length of 32 mm slightly narrows the low-frequency bandwidth to 0.473–0.792 GHz while introducing a new high-frequency resonance peak at 1.326–1.697 GHz, thereby substantially increasing the overall bandwidth. This observation confirms that the beveled meandering technique effectively excites additional resonance modes, representing a key factor in achieving broadband extension.

**Figure 5:**
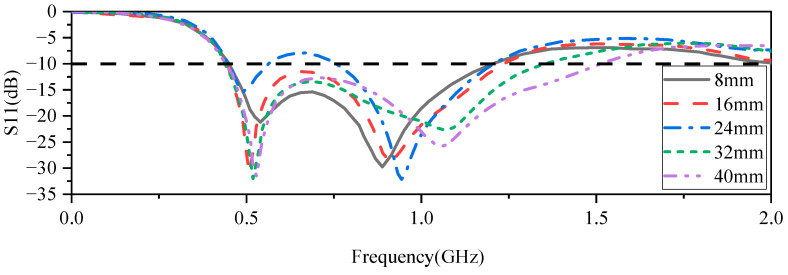
Ground plane truncation lengths on the reflection coefficient of microstrip antennas.

With the bevel length of the radiating patch maintained at 32 mm, right-angled triangular bevels were further introduced at the upper-left and upper-right corners of the ground plane; the corresponding simulation results are presented in Figure 5. Compared with the configuration where only the patch was beveled (Figure 4, bandwidths of 0.473–0.792 GHz and 1.326–1.697 GHz), a ground-plane bevel length of 40 mm shifts the lower edge of the low-frequency band from 0.473 GHz down to 0.442 GHz, and the two resonance peaks converge and merge, yielding a continuous bandwidth of 0.442–1.515 GHz. These results confirm that beveling the ground plane improves impedance matching and promotes the fusion of multiple resonant modes.

**Figure 6:**
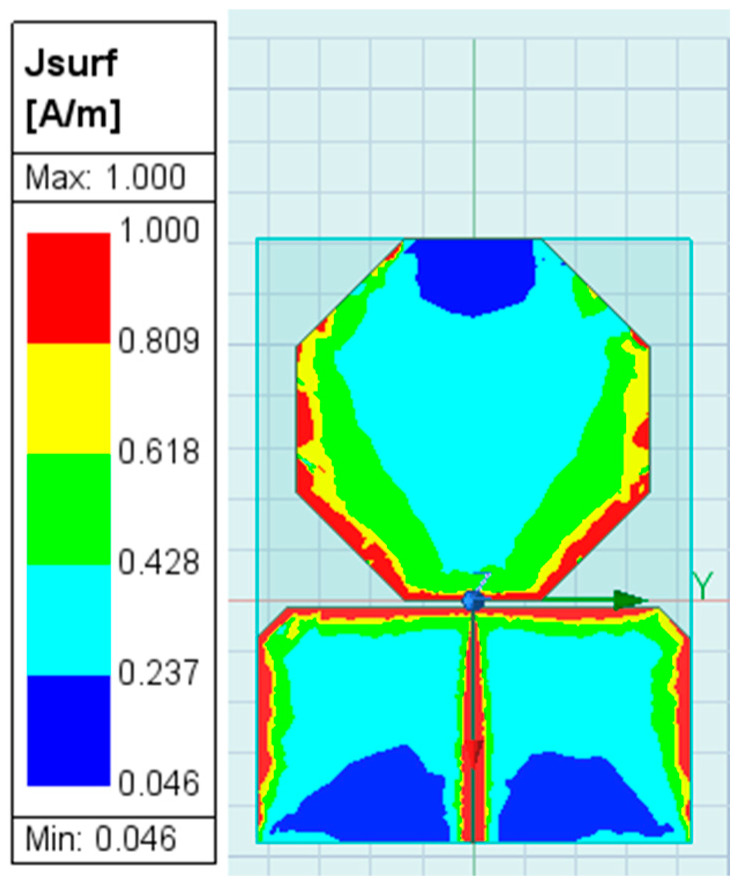
Surface current distribution of the antenna at 1.0 GHz.

To further verify the influence of the partial ground plane and the beveled meandering structure on the current path, Figure 6 presents the surface current distribution of the antenna at 1.0 GHz. As illustrated, the surface current is primarily concentrated along the feed line, at the edges of the radiating patch near the ground plane, and at the edges of the ground plane near the patch. The current density is highest in the feed line region. After propagating along the feed line to the patch edges, part of the current couples to the ground plane, indicating that the partial ground plane concentrates energy at the patch–ground plane interface. Meanwhile, the beveled structure effectively modifies the current path and excites additional resonance modes. These current distribution characteristics confirm the effectiveness of the proposed design in broadening the bandwidth.

## 3. Optimization of the Microstrip Sensor Design

The bandwidth performance of a flexible microstrip antenna is governed by the parameters of the radiating patch, the ground plane, and the meandering structure. Conventional optimization methods typically rely on adjusting a single structural parameter, failing to adequately account for the coupling effects among multiple parameters. Consequently, achieving both broadband performance and miniaturization simultaneously is challenging. To address this issue, this study proposes a multi-parameter co-optimization approach that employs an improved whale optimization algorithm (I-WOA) integrating multiple strategies for global optimization. This approach prioritizes the maximization of operating bandwidth while concurrently minimizing the structural dimensions.

### 3.1. Whale Optimization Algorithm Improved with Multiple Strategies

The whale optimization algorithm (WOA) is inspired by the hunting strategies of humpback whales, wherein each “whale” exhibits three distinct behavioral patterns: encircling prey, bubble-net attacking, and searching for prey. The algorithm is characterized by a small number of parameters, straightforward implementation, and a sufficiently broad search space. Nevertheless, WOA suffers from several limitations, including non-uniform distribution of initial solutions, slow convergence, and an inadequate trade-off between global exploration and local exploitation.

To address the aforementioned limitations of the WOA, this study introduces the Sobol sequence, leveraging its uniform sampling mechanism to enhance solution uniformity and diversity [17]. Furthermore, a dynamic inertia weight factor is incorporated to refine the mathematical models for the encircling-prey and searching-for-prey phases [18]. The specific expression of the adaptive inertia weight is given as follows:

(1) $$\omega (t)=1-\ln\left[1+\frac{(e-1)t}{t_{max}}\right]$$

In Equation (1), *t* denotes the current iteration count, and *t_max_* is the maximum number of iterations. As can be observed, the inertia weight *ω* is large during the early iterations, thereby enhancing the algorithm’s global exploration capability. As the iteration proceeds, *ω* gradually decreases and approaches zero, which shifts the algorithm’s focus toward local exploitation.

Finally, a Q-learning strategy is introduced to dynamically adjust individual behavior, following the approach of [19]. This method constructs a state–action reward table and a Q-table. Agents accumulate experience by iteratively trying different actions, comparing fitness values before and after each action, and updating the Q-table using the Bellman equation. During the early optimization stage, the optimal action is selected by comparing the Q-values associated with exploration and exploitation. The Bellman equation is expressed as follows:

(2) $$Q_{\left(t+1\right)}\left(s_{t},a_{t}\right)=Q_{\left(t\right)}\left(s_{t},a_{t}\right)+\lambda \left[r_{t+1}+\gamma MaxQ_{t}\left(s_{t+1},a_{t}\right)-Q_{t}\left(s_{t},a_{t}\right)\right]$$

In this formulation, *s_t_* and *s_t_*_+1_ denote the current and next states, respectively, while *a_t_* represents the action taken at iteration *t*. The learning rate *λ* controls the extent to which new information overrides existing knowledge and is set to 0.2. The discount factor *γ*, which balances immediate and future rewards, is set to 0.8. The term *r_t_*_+1_ is the immediate reward (or penalty) received by the agent after executing an action, and *Q*_(*t*+1)_ is the Q-value estimated in advance for the state *s_t_*_+1_. By incorporating Q-learning to account for the specific state of each whale agent, the algorithm’s global optimization capability in complex search spaces is significantly enhanced.

### 3.2. Multi-Dimensional Parameter Optimization Based on the Improved Whale Optimization Algorithm

The algorithm defines six geometric parameters as search variables: the length (*L_p_*) and width (*W_p_*) of the radiating patch, the length (*L_s_*) and width (*W_s_*) of the dielectric substrate, the patch bevel length (*L_f_*), and the ground-plane bevel length (*L_c_*). The following geometric constraints apply: *L_p_ ≤ L_s_*, *L_f_ ≤ W_p_/*2, *W_p_ ≤ W_s_* and *L_c_ ≤ L_b_*, where *L_b_* = *L_s_* − *L_p_* – 3 mm ensures a minimum clearance between the feed line and the ground plane. The optimization objectives are to maximize the operating bandwidth and minimize the antenna size. A global optimization strategy is employed for the concurrent design of the flexible microstrip antenna’s structural dimensions, with the following fitness function:

(3) $$fitness=\frac{f_{\max}}{f}+0.1\times \frac{S}{S_{\max}}$$

In Equation (3), the constraints are *f* ≤ *f_max_* (*f_max_* = 2 GHz), *S* ≤ *S_max_* (*S_max_* = 190 × 280 mm). The bandwidth term dominates the head of the power-law decay curve, where increasing bandwidth effectively improves convergence, whereas the area term lies in the tail, where reducing area has limited impact. Hence, a weight of 0.1 is assigned to the area term to emphasize the bandwidth objective.

**Figure 7:**
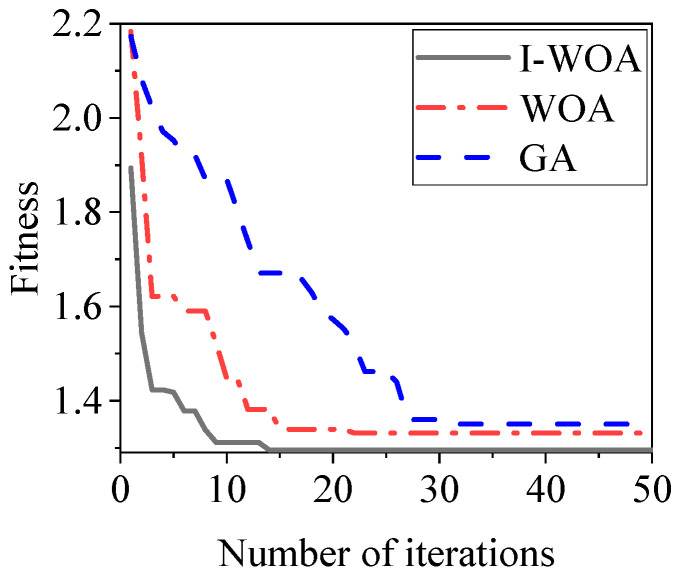
Convergence curves of standard GA, standard WOA, and I-WOA.

To validate the optimization performance of the I-WOA, it was compared against the standard WOA and a genetic algorithm (GA). Figure 7 presents the global best fitness iteration curves for the three algorithms. As illustrated, the GA exhibits multiple plateaus during optimization, converges slowly, and ultimately reaches a fitness value of 1.3504. The standard WOA converges faster than the GA, though its final fitness value remains slightly higher than that of the I-WOA. In contrast, the I-WOA outperforms both algorithms in terms of initial solution quality, convergence speed, and final fitness value, attaining a value of 1.2957. These results demonstrate that the I-WOA can identify optimal structural parameters more efficiently, thereby achieving simultaneous bandwidth maximization and size minimization.

**Figure 8:**
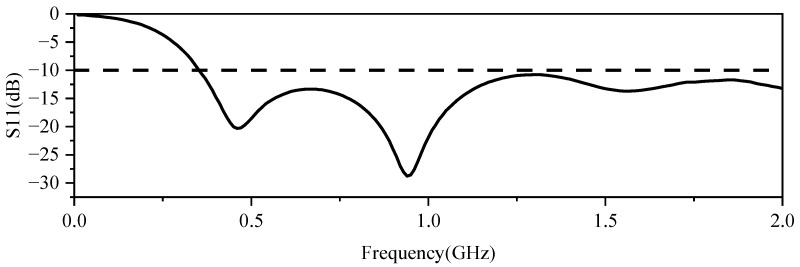
Simulated reflection coefficient of the novel flexible microstrip antenna.

The I-WOA optimization yielded a final fitness value of 1.2957 with the following optimized parameters: *Lp* = 141.56 mm, *Wp* = 138.24 mm, *Ls* = 236.70 mm, *Ws* = 169.46 mm, *Lf* = 42.25 mm, and *Lc* = 11.66 mm. The resulting dimensions of the optimized flexible microstrip antenna are summarized in Table 2, and the corresponding simulated return loss curve is presented in Figure 8. The optimization was carried out with a population size of 50 and 50 iterations, requiring 2500 HFSS simulations in total. This computational cost is acceptable for offline antenna optimization and can be mitigated by parallel simulation or surrogate modeling in future work.

As shown in Table 2 and Figure 8, the optimized flexible microstrip antenna achieves a compact size of 236.70 × 169.46 × 0.25 mm, corresponding to a 24.7% reduction (i.e., 75.3% of its original dimensions). The operating bandwidth is expanded to 0.361–2.0 GHz, covering the standard UHF PD detection range for transformers (500–1500 MHz) and partially encompassing the RF detection range for cable joints (10–600 MHz). Notably, this design maintains a compact footprint while delivering superior bandwidth performance.

It should be noted that the bandwidth expansion entails a slight degradation of the antenna’s radiation performance in the low-frequency range. Nevertheless, as this frequency range is not the primary band for partial discharge detection, the overall detection performance of the sensor remains sufficient for monitoring purposes, rendering this trade-off acceptable.

**Figure 9:**
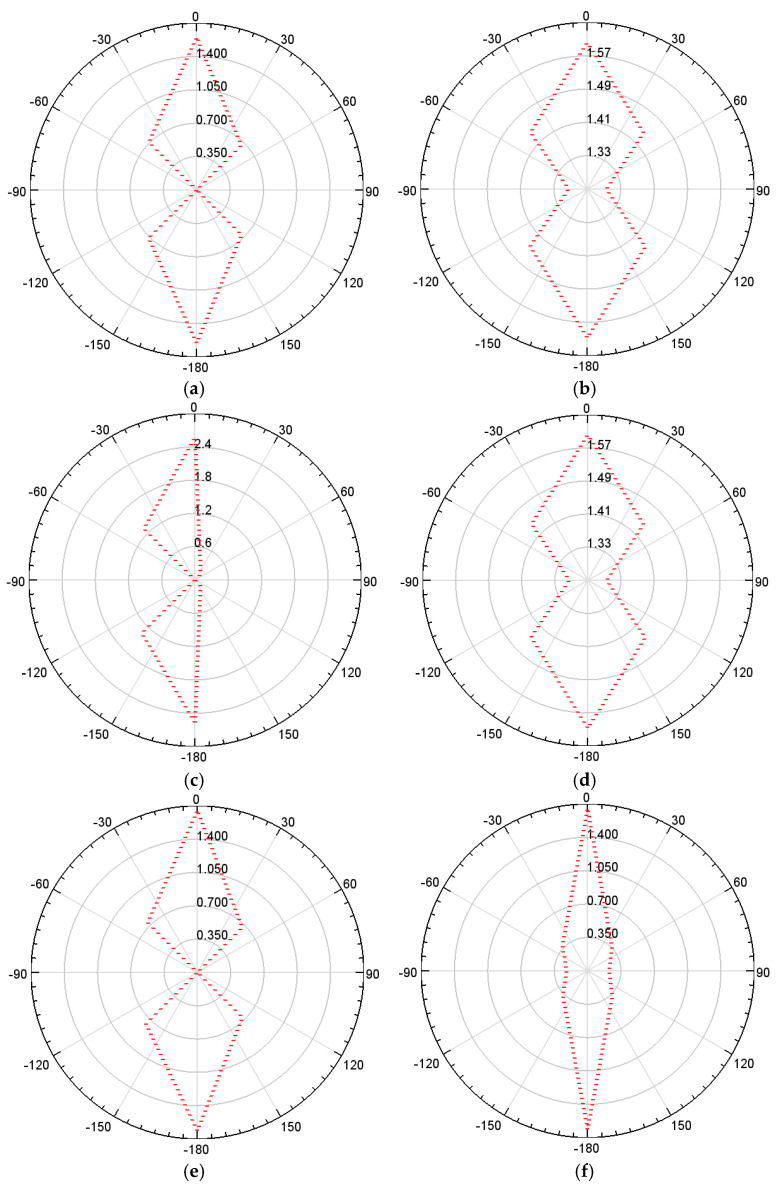
Simulated E-plane (*Φ* = 0°) and H-plane (*Φ* = 90°) radiation patterns of the antenna at 0.5 GHz, 1.0 GHz, and 1.5 GHz: (**a**) 0.5 GHz, E-plane; (**b**) 0.5 GHz, H-plane; (**c**) 1.0 GHz, E-plane; (**d**) 1.0 GHz, H-plane; (**e**) 1.5 GHz, E-plane; (**f**) 1.5 GHz, H-plane.

To comprehensively evaluate the radiation characteristics of the designed antenna, far-field simulations of the optimized antenna were conducted using HFSS. Table 3 summarizes the simulated gain values at selected frequencies within the operating band, and Figure 9 presents the simulated E-plane (*Φ* = 0°) and H-plane (*Φ* = 90°) radiation patterns at three representative frequencies: 0.5 GHz, 1.0 GHz, and 1.5 GHz.

As shown in Table 3, the simulated gains of the antenna at 0.5 GHz, 1.0 GHz, and 1.5 GHz are 1.60 dBi, 2.45 dBi, and 1.80 dBi, respectively. The peak gain occurs near 1.25 GHz, reaching approximately 2.55 dBi. The gain initially increases with frequency and then decreases, which is consistent with the typical gain variation pattern observed in microstrip antennas.

As shown in Figure 9, the antenna exhibits favorable radiation characteristics across the measured frequency range. The E-plane patterns are approximately figure-eight-shaped at all three frequencies, whereas the H-plane patterns are roughly elliptical, indicating good omnidirectional behavior in the azimuth plane. The E-plane patterns display slight front-to-back asymmetry, which is typical for microstrip antennas employing a partial ground plane. The gain values extracted from these patterns are consistent with the simulated gains reported in Table 3. Overall, the antenna maintains satisfactory radiation performance at 0.5 GHz, 1.0 GHz, and 1.5 GHz.

**Figure 10:**
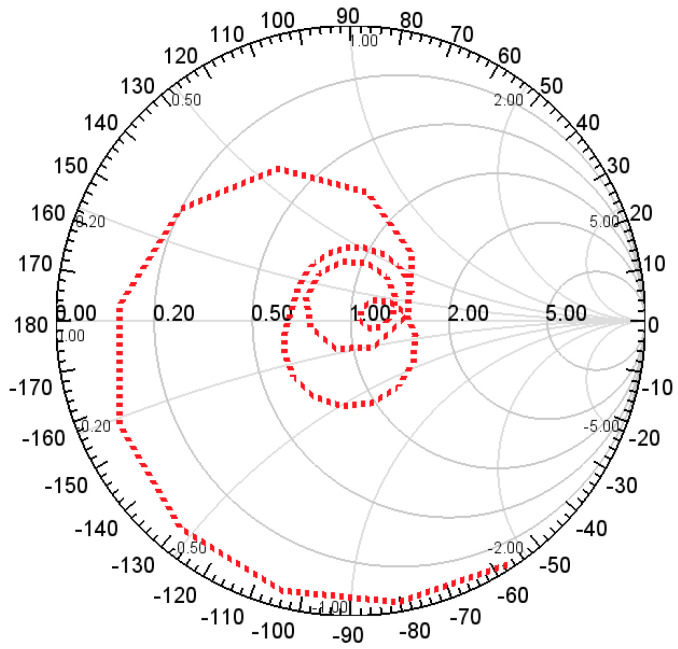
Simulated Smith chart of the antenna input impedance (0.1–2.0 GHz).

To evaluate the antenna’s broadband impedance matching characteristics, Figure 10 presents a simulated Smith chart of the antenna’s input impedance over the range of 0.1–2.0 GHz. As shown, the S(1,1) trace begins in the low-frequency region, spirals toward the center of the chart as the frequency increases, and forms multiple small loops across the entire operating band. This behavior indicates that the antenna excites multiple resonance modes, thereby achieving broadband impedance matching. These results validate the effectiveness of the partial ground plane and the beveled meandering structure in extending the bandwidth.

## 4. Evaluation of the Novel Sensor with Reflection Coefficient Measurements and a GTEM Cell

**Figure 11:**
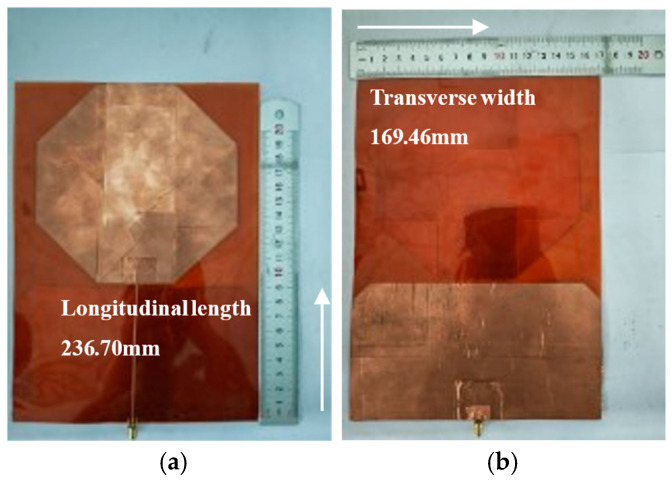
Fabricated prototype of the novel flexible microstrip antenna: (**a**) front view; (**b**) back view.

Based on the optimized dimensional parameters, a prototype was fabricated using a flexible polyimide substrate as the dielectric material, with the radiating patch and ground plane made of 0.065 mm thick copper foil. The resulting antenna is shown in Figure 11.

To evaluate the bandwidth characteristics of the novel flexible microstrip antenna and its sensitivity for partial discharge detection, both bandwidth and sensitivity tests were conducted on the fabricated prototype.

### 4.1. Reflection Coefficient Measurement and Analysis

**Figure 12:**
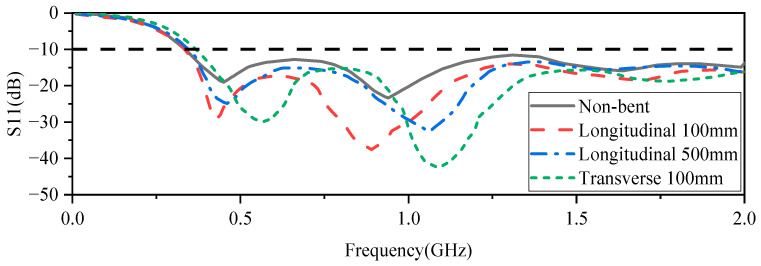
Measured reflection coefficient of the novel flexible microstrip antenna.

A spectrum analyzer with a 2 GHz bandwidth and network analysis functionality was used to measure the return loss of the antenna under both flat and bent conditions, with bending radii of 100 mm and 500 mm. The results are presented in Figure 12. In the flat state, the bandwidth was 0.346–2 GHz. When bent along the length direction with a radius of 100 mm, the bandwidth was 0.363–2 GHz; with a radius of 500 mm, it was 0.357–2 GHz. When bent along the width direction with a radius of 100 mm, the bandwidth was 0.336–2 GHz; with a radius of 500 mm, it was 0.341–2 GHz. Compared with the simulated lower cutoff frequency (0.361 GHz) shown in Figure 8, the measured value for the flat antenna (0.346 GHz) is slightly lower. Possible sources of this discrepancy include manufacturing tolerances, fabrication processes, and the influence of the test setup and environment. The results also indicate that the antenna bandwidth varies under different bending conditions. Specifically, bending along the length direction raises the lower cutoff frequency by approximately 3–5%, whereas bending along the width direction lowers it by approximately 1–3%. These fluctuations are likely attributable to changes in stray capacitance and inductance induced by antenna bending. In addition to manufacturing and environmental factors, the HFSS simulation itself introduces certain deviations. The simulation model assumes an ideal planar antenna structure, employing perfect conductive boundaries and uniform dielectric parameters, while neglecting the actual surface roughness of the copper foil, the frequency dependence of the polyimide dielectric constant, and the parasitic parameters introduced by the SMA connector. Moreover, HFSS’s finite-element meshing and adaptive convergence criteria also affect simulation accuracy. Collectively, these factors contribute to the discrepancies observed between the simulated and experimental results.

It should be noted that the polyimide substrate employed in this study exhibits a certain degree of hygroscopicity. In high-humidity environments, the dielectric constant of the substrate may undergo slight variations, potentially causing a drift in the antenna’s resonance frequency. However, because the novel sensor operates over a wide frequency band (0.346–2.0 GHz) and the frequency accuracy requirements for partial discharge detection are relatively lenient, any humidity-induced frequency shift is not expected to significantly affect detection performance. Dedicated humidity impact tests will be conducted in future work to further validate this.

### 4.2. GTEM Cell-Based Sensitivity Calibration

**Figure 13:**
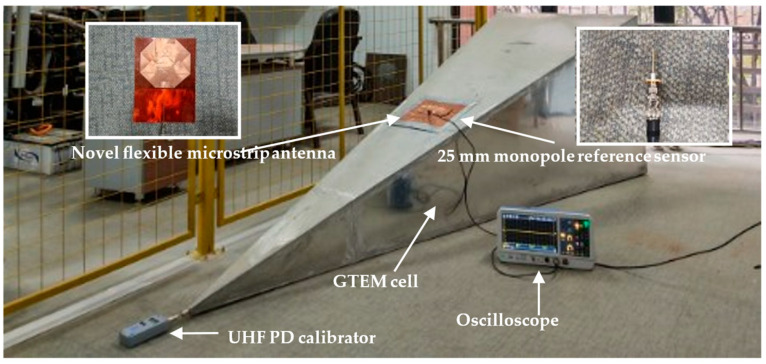
Experimental testbed for sensitivity calibration.

**Figure 14:**
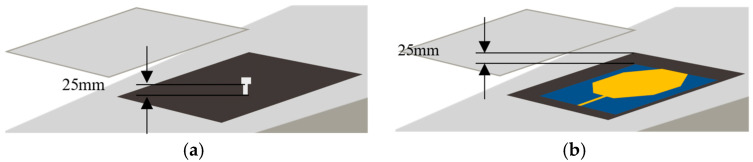
Partial enlarged view of the GTEM cell measurement window: (**a**) reference monopole probe; (**b**) flexible microstrip antenna.

The sensitivity of an RF partial discharge sensor can be expressed in terms of effective height, defined as the ratio of the sensor’s output voltage amplitude to the incident electric field amplitude at each frequency, with units of millimeters (mm). The effective height can be determined using a gigahertz transverse electromagnetic (GTEM) cell. Figure 13 shows the GTEM cell used in this study (dimensions: 3 × 1 × 1 m), which features a 0.3 × 0.3 m square aperture on its top that serves as the measurement window [20]. The test procedure is as follows. First, a 25 mm monopole probe, acting as the reference antenna, is placed inside the GTEM cell measurement window, and its output voltage response *U_Mr_* is acquired. It should be noted that the photograph in Figure 13 shows the sensors alone for illustration purposes and does not represent their actual installation position, and Figure 14 shows the sensors placed inside the measurement window with the shielding cover open. During actual measurements, the measurement window is covered with a shielding cover to ensure proper electromagnetic isolation. Using the known effective height *H_ref_* of the reference antenna, the effective height *H_sens_* of the antenna under test can be calculated according to Equation (4) [21].

(4) $$H_{sens}=\frac{U_{Ms}}{U_{Mr}}H_{ref}$$

**Figure 15:**
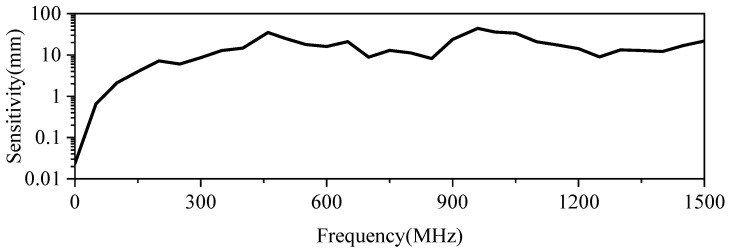
Sensitivity calibration results of the novel flexible microstrip antenna.

In the measurements, a pulse generator with a rise time of approximately 350 ps served as the input to the GTEM cell, and an oscilloscope with a sampling rate of 5 GS/s was used to record the sensor output. To minimize random errors, each set of experiments was repeated five times, and the results are expressed as “mean ± standard deviation.” Taking the 500 MHz frequency point as an example, the measured effective height was 25.49 ± 0.52 mm (*n* = 5), indicating good measurement repeatability. The sensitivity test results of the novel flexible microstrip antenna are presented in Figure 15. Between 0 and 289 MHz, the average sensitivity is below 8 mm. However, when the frequency exceeds 289 MHz, the minimum sensitivity values all exceed 8 mm, with an average of 18.77 mm. Thus, within the 289–1500 MHz frequency band, the antenna’s sensitivity complies with the requirement of the State Grid of China standard Q/GDW 11311-2014, which stipulates that the average effective height of a sensor within its operating frequency band shall not be less than 8 mm [22]. Moreover, this frequency band effectively covers the ultra-high-frequency (UHF) detection range for partial discharges in transformers (500–1500 MHz) and also encompasses the latter part of the radio-frequency (RF) detection range for partial discharges in cable joints (10–600 MHz).

It should be noted that the aforementioned sensitivity tests (effective height) were performed with the antenna in a flat configuration. For bent configurations, systematic sensitivity testing is technically challenging due to the absence of standardized calibration methods for bent antennas; consequently, such tests were not conducted in this study. Nevertheless, the observed increase in response amplitude under bent conditions (Section 5.2) directly demonstrates the practical advantage of flexible antennas on curved surfaces. A comprehensive sensitivity uncertainty analysis for bent configurations will be undertaken in future work.

## 5. Evaluation of the Novel Sensor via Measuring PD Calibration Pulses

To evaluate the practical performance of the developed flexible microstrip antenna in partial discharge detection, a partial discharge calibration pulse generator was used to simulate a discharge source. The antenna’s detection capability was tested under both transformer and cable joint partial discharge scenarios.

### 5.1. Transformer PD Detection Test

**Figure 16:**
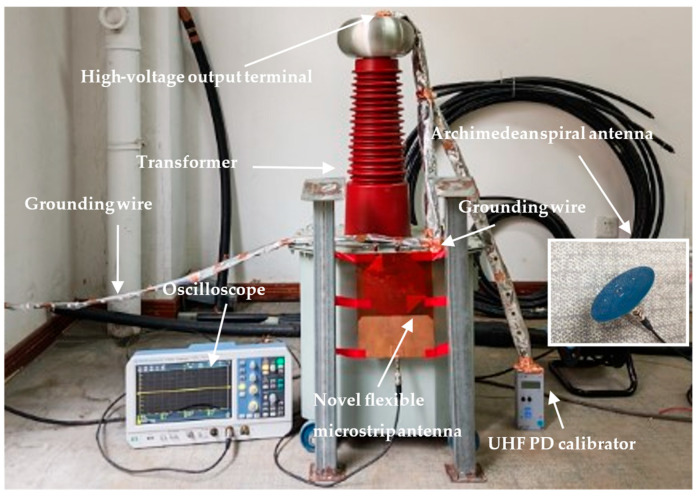
Experimental testbed for transformer PD detection.

**Figure 17:**
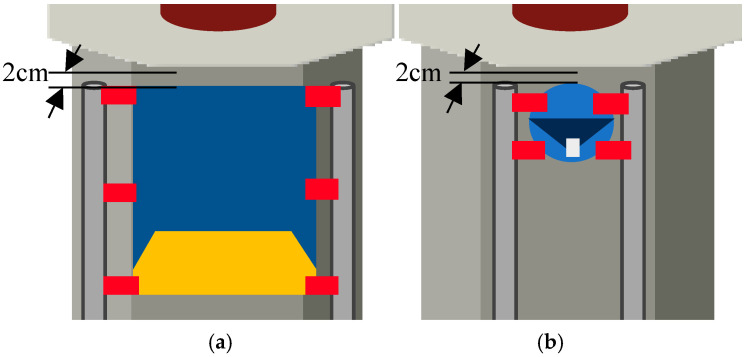
Partial enlarged views of sensor placement on the transformer tank: (**a**) Novel flexible microstrip antenna. (**b**) Archimedean spiral antenna.

**Figure 18:**
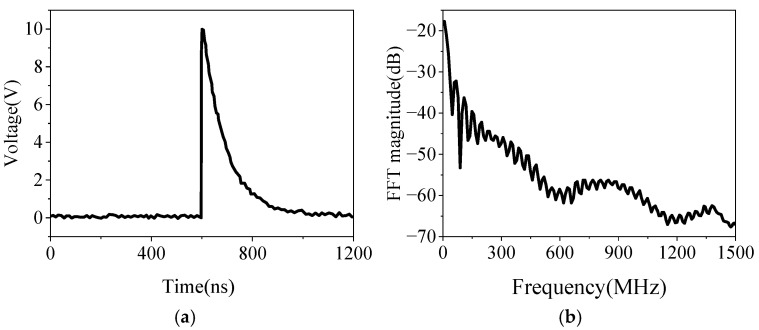
Waveform and frequency spectrum of the 200 ps rise time excitation source. (**a**) Time domain waveform. (**b**) Frequency spectrum.

The test platform for transformer partial discharge calibration pulse detection is shown in Figure 16. The output of a UHF partial discharge calibrator was connected to the high-voltage and ground terminals of a test transformer (100 kV/10 kVA), and calibration pulses were injected to simulate partial discharges within the transformer. As illustrated in Figure 17, the radiating surface of the flexible microstrip antenna was oriented toward the transformer and positioned at the top region near the junction of the transformer tank and the high-voltage bushing, close to but not touching the outer tank wall (with a gap of approximately 2 cm). The antenna output was connected to an oscilloscope with a sampling rate of 5 GSa/s. The UHF partial discharge calibrator was capable of generating pulse signals with voltage amplitudes ranging from 2 V to 50 V and a rise time of 200 ps. Figure 18 presents the measured time-domain waveform and frequency spectrum of the output pulse at an amplitude of 10 V. The connecting cable between the calibrator and the transformer was shielded to minimize electromagnetic interference from cable leakage.

Notably, because Archimedean spiral antennas have been successfully applied in transformer partial discharge detection [23], such an antenna (outer diameter: 100 mm, feed line length: 40 mm) was selected as a reference for comparison and installed at the same location, as shown in Figure 17. To confirm that the acquired signals were genuine partial discharge pulses rather than environmental electromagnetic interference or antenna self-resonance, background noise was recorded without injecting calibration pulses, and the SNR was calculated using Equation (5).

(5) $$SNR=20log10\;\left(\frac{U_{signal}\;}{U_{noise}}\right)$$

where *U_signal_* and *U_noise_* denote the amplitudes of the discharge signal and the background noise, respectively.

**Figure 19:**
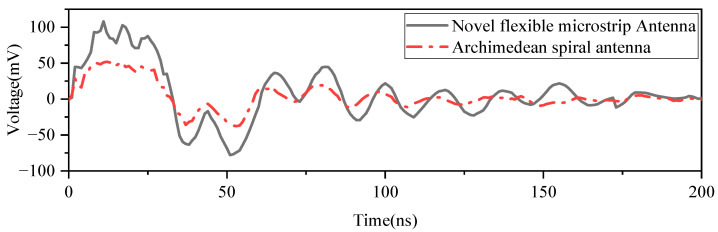
Time-domain detection results of PD in transformers.

**Figure 20:**
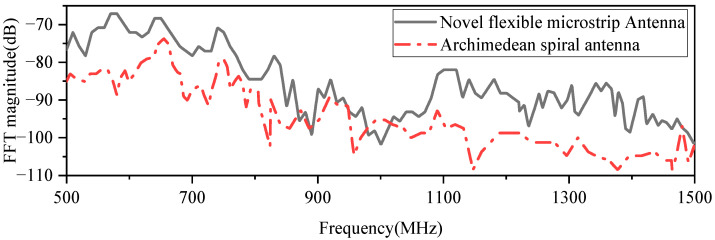
FFT spectrum detection results of PD in transformers.

During the experiment, the calibration pulse amplitude was gradually increased from 2 V to 50 V, and the response voltages of both sensors were recorded. The results showed that the response voltage amplitudes of the two sensors were proportional to the calibration pulse amplitude, while the voltage waveforms remained unchanged. Figure 19 presents the signals measured by the two sensors at a calibration pulse amplitude of 10 V. After five repeated measurements, the output signal amplitude of the novel flexible microstrip antenna was 107.94 ± 2.1 mV (n = 5), with a background noise amplitude of 0.40 ± 0.03 mV (n = 5) and an SNR of 48.62 ± 0.5 dB (n = 5). In comparison, the Archimedean spiral antenna yielded an output signal amplitude of 51.92 ± 1.5 mV (n = 5), a background noise amplitude of 0.90 ± 0.05 mV (n = 5), and an SNR of 35.22 ± 0.6 dB (n = 5). These results demonstrate that the novel antenna exhibits a higher discharge response amplitude, lower noise level, and significantly better SNR. The frequency spectra of the signals acquired by the two sensors are shown in Figure 20. Evidently, except for a few frequency points, the response sensitivity of the novel flexible microstrip antenna surpasses that of the Archimedean spiral antenna.

### 5.2. Cable Joint PD Detection Test

**Figure 21:**
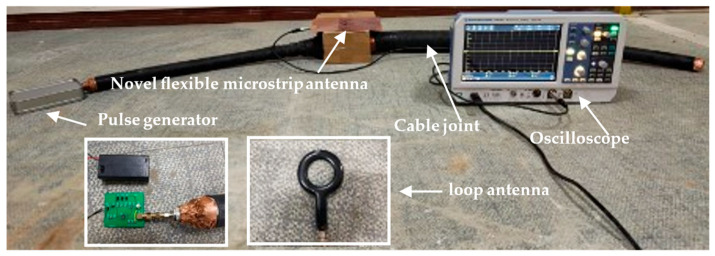
Experimental testbed for cable joint PD detection.

**Figure 22:**
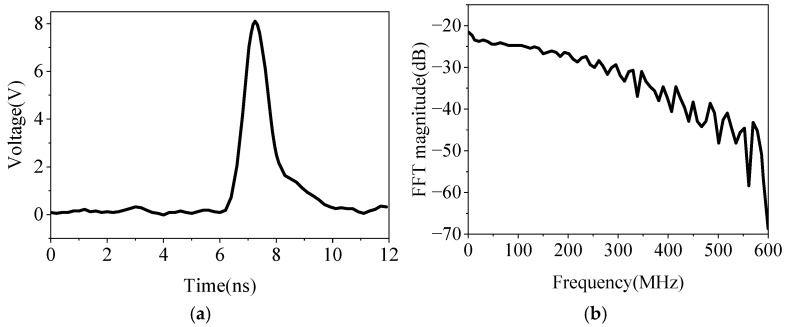
Waveform and frequency spectrum of the 890 ps rise time excitation source: (**a**) time domain waveform; (**b**) frequency spectrum.

The test platform for calibration pulse detection of partial discharges in cable joints is shown in Figure 21. The cable joint sample, with a total length of approximately 3 m, consisted of a 35 kV single-core XLPE cable and a prefabricated cold-shrink joint. One end of the joint sample was left open, while the other end was connected to the calibration pulse generator. Both ends of the joint sample were wrapped in copper foil, and the pulse generator circuitry was enclosed in an aluminum box to suppress electromagnetic signal leakage from these locations and minimize its impact on the experimental results. Furthermore, considering that the frequency range for RF detection of partial discharges in cable joints is primarily below 600 MHz, the calibration pulse generator used in this test was capable of outputting pulses with a rise time of 890 ps and an amplitude of 8 V. The time-domain waveform and frequency spectrum of the pulse are shown in Figure 22. For comparison, in addition to the Archimedean spiral antenna used in the transformer tests, a loop antenna (with outer and inner diameters of 60 mm and 35 mm, respectively) was also introduced. This loop antenna has been previously applied in RF detection of partial discharges in cable joints [24].

**Figure 23:**
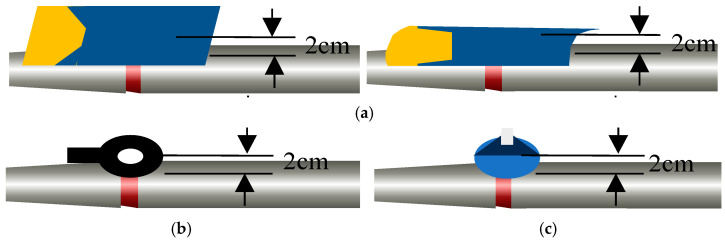
Partial enlarged views of sensor placement on the cable joint: (**a**) Novel flexible microstrip antenna. (**b**) Loop antenna. (**c**) Archimedean spiral antenna.

**Figure 24:**
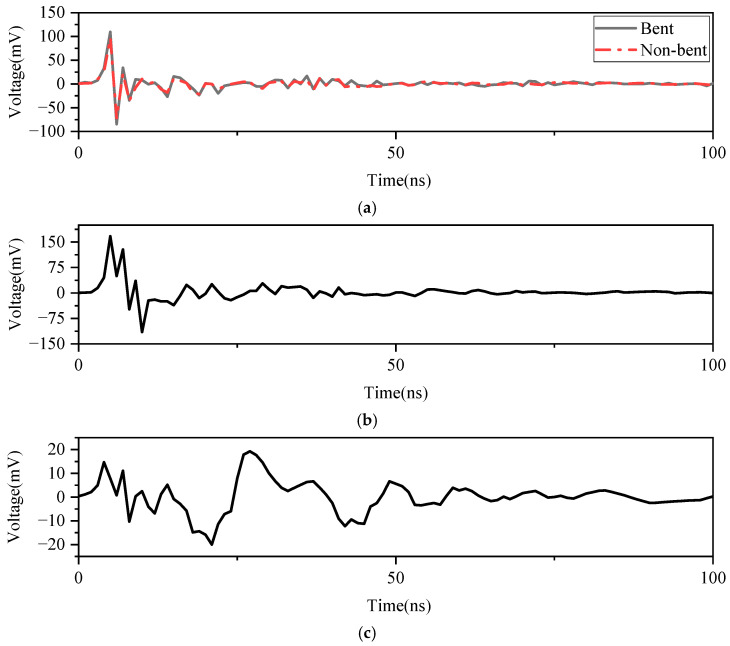
Time-domain detection results of PD in cable joints. (**a**) Novel flexible microstrip antenna. (**b**) Loop antenna. (**c**) Archimedean spiral antenna.

**Figure 25:**
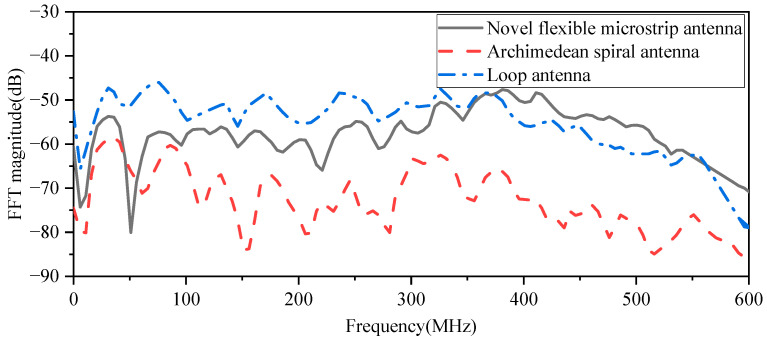
FFT spectrum detection results of PD in cable joint.

During the experiment, the three sensors were placed at the same position on the joint sample, close to but not touching the joint surface (with a gap of approximately 2 cm), as illustrated in Figure 23. To evaluate the performance of the flexible microstrip antenna in a bent state, measurements were conducted with the antenna both flat and bent (wrapped around the joint along its axial centerline). Figure 24 presents the measurement results for the three sensors. After five repeated measurements, the output signal amplitude of the novel flexible microstrip antenna in the flat state was 96.37 ± 1.8 mV (n = 5), compared to 168.0 ± 2.5 mV (n = 5) for the loop antenna and 20.6 ± 0.6 mV (n = 5) for the Archimedean spiral antenna. When the novel antenna was bent to wrap around the cable joint, its output signal amplitude increased to 109.79 ± 2.0 mV (n = 5), corresponding to an improvement of approximately 14% relative to the flat configuration. The frequency spectra of the signals measured by the three sensors are shown in Figure 25. The loop antenna exhibits the highest response sensitivity below 370 MHz, whereas the novel flexible microstrip antenna demonstrates the highest sensitivity above 370 MHz.

## 6. Conclusions

This paper has addressed the inherent trade-off between bandwidth expansion and size minimization in microstrip antennas for RF detection of partial discharges in electrical equipment. Based on a flexible microstrip antenna, structural improvements and performance enhancements were investigated, leading to a novel flexible microstrip antenna design. A prototype was fabricated according to this design, and its performance was experimentally validated. The main conclusions are as follows:

1. By incorporating a partial ground plane and an oblique-cut meandering technique, and by optimizing the structural parameters of the microstrip antenna using an improved whale optimization algorithm (I-WOA), a novel flexible microstrip antenna was developed. The antenna achieves an operating bandwidth of 0.346–2.0 GHz and dimensions of 236.70 × 169.46 × 0.25 mm. Moreover, the variation in the lower cutoff frequency of the antenna remains within 5% under different bending conditions.
2. Measurements conducted in the GTEM cell show that the antenna achieves an average effective height (sensitivity) of 18.77 mm in the 289–1500 MHz frequency range, fully complying with the requirements of the relevant industry standards.
3. The effectiveness of the novel antenna for RF partial discharge detection was further validated through calibration pulse detection tests. Notably, in the cable joint tests, bending the antenna to conformally wrap around the joint increased the response amplitude by 14% compared with the unbent configuration.

Despite the achievements of this work, the proposed flexible microstrip antenna still exhibits lower response performance near the lower cutoff frequency of its bandwidth compared with the reference loop antenna. Future work will focus on further refining the sensor design and optimization algorithms to enhance performance in this frequency range. It should also be acknowledged that the current antenna dimensions are not suitable for very small-scale high-voltage equipment. Further miniaturization will be pursued in future work for applications requiring smaller form factors.

## Figures and Tables

**Table 1:** Dimensions of the flexible microstrip antenna.

Parameter	*Ls*	*Ws*	*Lp*	*Wp*	*Lb*	*Wb*	*Wf*	*h*
Size (mm)	190	280	130	220	190	280	0.5	0.25

**Table 2:** Dimensions of the novel flexible microstrip antenna.

Parameter	*Ls*	*Ws*	*Lp*	*Wp*	*Lf*
Size (mm)	236.70	169.46	141.56	138.24	42.25
Parameter	*Wf*	*Lb*	*Wb*	*Lc*	*h*
Size (mm)	0.5	92.14	169.46	11.66	0.25

**Table 3:** Simulated gain of the antenna at representative frequencies.

Frequency (GHz)	0.5	1.0	1.5
Gain (dBi)	1.60	2.45	1.80

## Data Availability

The data presented in this study are available on request from the corresponding author due to privacy restrictions.
